# Insulin Allergy to Detemir Followed by Rapid Onset of Diabetic Ketoacidosis: A Case Report and Literature Review

**DOI:** 10.3389/fendo.2022.844040

**Published:** 2022-03-08

**Authors:** Chong Boon Teo, Pek Yan Tan, Shan Xian Lee, Joan Khoo, Jun Guan Tan, Su Fen Ang, Sze Hwa Tan, Tunn Lin Tay, Eberta Tan, Su Chi Lim, Bernhard O. Boehm, Wann Jia Loh

**Affiliations:** ^1^ Yong Loo Lin School of Medicine, National University of Singapore, Singapore, Singapore; ^2^ Department of Dermatology, Changi General Hospital, Singapore, Singapore; ^3^ Department of Endocrinology, Changi General Hospital, Singapore, Singapore; ^4^ Department of Laboratory Medicine, Khoo Teck Puat Hospital, Singapore, Singapore; ^5^ Clinical Research Unit, Khoo Teck Puat Hospital, Singapore, Singapore; ^6^ Department of Laboratory Medicine, Changi General Hospital, Singapore, Singapore; ^7^ Diabetes Centre, Admiralty Medical Centre, Singapore, Singapore; ^8^ Department of Endocrinology, Tan Tock Seng Hospital, Singapore, Singapore; ^9^ Lee Kong Chian School of Medicine, Nanyang Technological University Singapore, Singapore, Singapore; ^10^ Type 1 Diabetes Genetics Consortium (T1DGC) European Repository, Ulm University, Ulm, Germany

**Keywords:** insulin allergy, insulin hypersensitivity, insulin, insulin-dependent diabetes, diabetic ketoacidosis

## Abstract

The management of diabetes mellitus in an insulin-dependent patient is challenging in the setting of concomitant antibody-mediated-insulin hypersensitivity. We report a case of a 62-year-old woman with pre-existing type 2 diabetes mellitus of 10 years duration who developed type 3 hypersensitivity reaction to insulin analogue detemir, and subsequently, severe diabetic ketoacidosis (DKA). She was C-peptide negative and was diagnosed with insulin-dependent diabetes. Despite increasing dose adjustments, insulin-meal matching, and compliance with insulin, she experienced episodes of unexpected hyperglycaemia and hypoglycaemia. The development of rash after detemir initiation and rapid progression to DKA suggests an aberrant immune response leading to the insulin allergy and antibody-induced interference with insulin analogues. Glycaemic control in the patient initially improved after being started on subcutaneous insulin infusion pump with reduced insulin requirements. However, after a year on pump therapy, localised insulin hypersensitivity reactions started, and glycaemic control gradually deteriorated.

## Introduction

Insulin therapy is necessary in the management of insulin deficient diabetes mellitus, but exogenous insulin can cause severe complications apart from hypoglycaemia. These lesser known but clinically meaningful complications include insulin allergy (1), immunological insulin resistance (2) and lipoatrophy (3). Prior to the advent of purification techniques, the use of insulin derived from porcine (which differs from human insulin in one carboxy-terminal amino acid of the B-chain, where alanine substitutes for threonine) and bovine (which differs from human insulin which has threonine and isoleucine whereas bovine insulin has alanine and valine at positions 8 and 10) led to frequent hypersensitivity reactions (1). True insulin allergy is currently an uncommon phenomenon with an estimated prevalence of <0.1-1% with a wide range of symptoms ranging from localised reactions to anaphylaxis (1, 4–6). We present here an unusual case of an adult female who shortly after insulin detemir (Levemir) initiation, developed type III hypersensitivity reaction, and was admitted for severe diabetic ketoacidosis (DKA) 1 week after stopping detemir. The management was challenging because of high insulin requirements and cutaneous hypersensitivity reactions to insulin. We also performed a literature review of patients with insulin-dependent diabetes with insulin allergy to inform our management of this rare condition.

## Case Description

**Figure 1:**
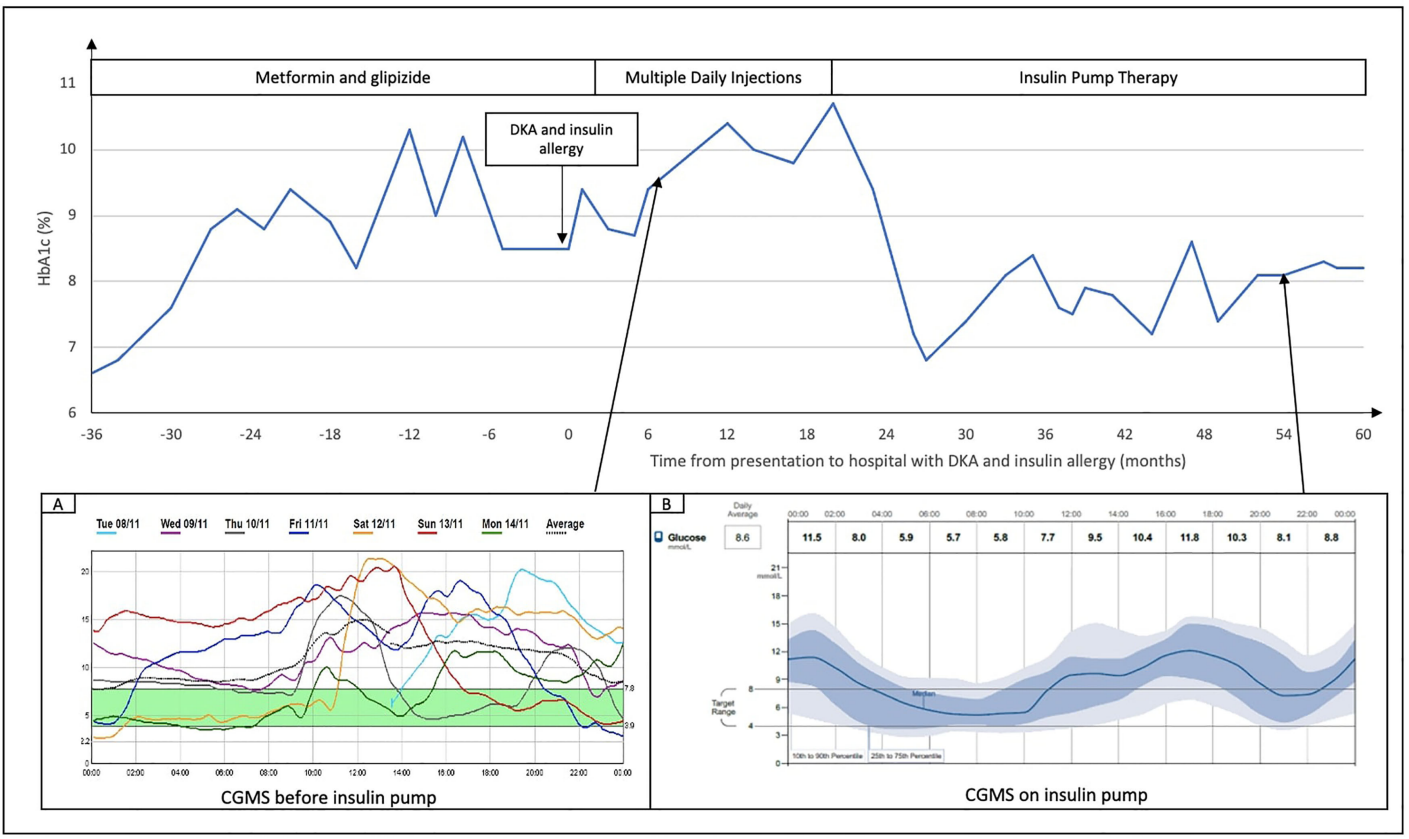
Glycaemic control in this patient, reflected as HbA1c trend, and continuous glucose monitoring (CGMS) before **(A)** and after **(B)** insulin pump. Time on X axis is presented as months from presentation to hospital with severe DKA and insulin (detemir) allergy.

A 62-year-old female of Chinese ethnicity with a 10 years’ history of type 2 diabetes mellitus (T2DM) presented to the emergency department with a few days history of polyuria and polydipsia, and was diagnosed with DKA. In the first 7 years of her T2DM history, her glycaemic control was satisfactory (HbA1c of 6.6-7.6%) on combination regimen of metformin and glipizide. In the recent 3 years, her glycaemic control gradually worsened to a peak HbA1c of 10% but this was controlled by intensifying diet compliance and increasing metformin and glipizide doses to maintain a HbA1c of 8-8.5% (**Figure 1**). Her general practitioner added insulin detemir as a basal insulin supplement 3 weeks prior to admission, following which she experienced pruritic erythematous rashes at the injection site within a day. She stopped the insulin injections after 2 weeks in view of intolerable localised rashes with minimal response to antihistamines and presented 1 week later with severe DKA. Her other past medical history included hypertension, hyperlipidaemia, and obesity (weight 70kg; BMI 27.5kg/m^2^). She did not have any known drug allergies, past medical history or family history of allergy and autoimmune disorders. Her mother had T2DM at old age.

**Figure 2:**
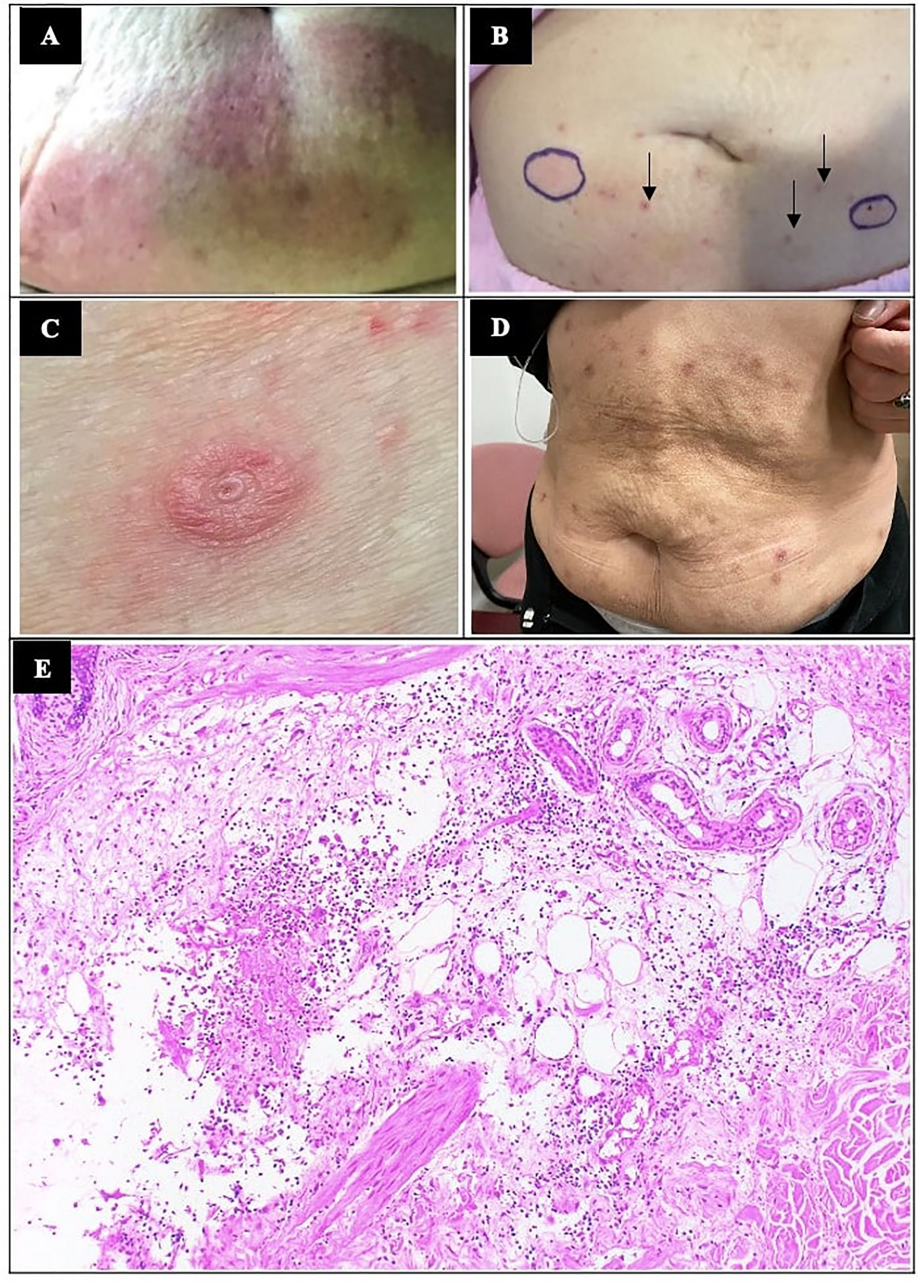
Localised cutaneous hypersensitivity reactions to insulininjections. **(A)** Localised erythematous rashes at sites of insulin detemir injections at initial presentation. **(B)** Localised erythematous skin reactions occurred after test doses of glulisine, circled in blue. A few of the resolving rashes (small arrows) at previous insulin detemir sites at 3 days after hospital admission are shown. **(C)** Localised cutaneous reaction at insulin administration sites when patient was on insulin aspart (novorapid) subcutaneous insulin pump is shown here. Patch testing to consumables of insulin pump were negative. **(D)** Increasingly altered cosmesis of her abdominal skin from repeated localised allergic reactions to insulin were observed after 4 years of insulin therapy. **(E)** Histology by haematoxylin and eosin staining (x100 magnification) showed dermal oedema and lymphohistiocytic perivascular inflammation suggestive of hypersensitivity reaction.

On clinical examination during her admission, there were extensive erythematous urticarial rashes at insulin injection sites on the abdomen (**Figure 2A**). Blood tests showed moderate DKA (hyperglycaemia of 24.7 mmol/L, ketonemia of 6.5 mmol/L, acidosis of pH 7.126 and serum venous bicarbonate of 6 mmol/L). Her serum glucose level was controlled at 10-14mmol/L and DKA resolved while on intravenous infusion of regular insulin (actrapid) without hypersensitivity reactions. Prior to converting fully to subcutaneous insulin, low doses of insulin analogues glulisine, glargine and aspart were injected subcutaneously to monitor for hypersensitivity; after a few hours, she developed localised erythematous rash to subcutaneous insulin glulisine (**Figure 2B**), which resolved after a few days, but not to insulin glargine and insulin aspart. The patient was discharged from hospital with multiple daily injection (MDI) therapy at 38 units of glargine once daily and 6-8 units of aspart pre-meals (preliminary estimation ICR 1: 6-8g), total daily dose [TDD] of 1 unit/kg/day, capillary glucose readings 5-18mmol/L). She was closely followed up at outpatient for insulin titration, however, her glycaemic control worsened despite compliance and increasing doses of basal and prandial insulin (**Figure 1**). Although she was now C-peptide negative, metformin was continued to reduce insulin resistance.

## Investigations

During her DKA, her full blood count, kidney function, liver function test, thyroid function test, lactate level, and procalcitonin level were all normal. There was no eosinophilia despite the allergic skin reactions and no evidence of infection with normal monocyte and leukocyte counts. Her HbA1c was 8.5%. Her vitamin levels such as folate, B12 and zinc levels were normal, except for vitamin D which was mildly deficient at 22.2 ug/L. Her retinal photo and urine albuminuria tests were normal. Apart from the insulin allergy to detemir, there were no clear precipitants to DKA such as infection, myocardial infarction, or pancreatitis. In view of the acute onset of complete insulin deficiency, evident by undetectable C-peptide levels, testing for pancreatic autoantibodies was performed. Her pancreatic autoantibodies were positive for islet cell antibody (anti-ICA) using indirect immunofluorescence against monkey pancreas, but negative for glutamic acid decarboxylase antibody (anti-GAD), IA2 and zinc transporter 8 antibody. Her C-peptide levels were repeatedly undetectable (<27pmol/L) when performed at presentation, few months after, and 2 years later, consistent with the diagnosis of complete insulin deficiency. Repeat anti-ICA and anti-GAD test were negative 2 years later.

Despite compliance and frequent insulin titrations, her glycemic control gradually deteriorated with unexpected hyperglycaemia and hypoglycaemia in the next 1 year warranting further investigations. CT pancreas was normal showing a pancreas of normal volume and with no structural lesions or inflammatory changes. She did not have any other autoimmune conditions such as Addison’s disease, Hashimoto thyroiditis (thyroid peroxidase antibody was negative and thyroid function test normal) or pernicious anaemia. She did not have clinical signs to suggest lipodystrophy syndrome or Cushing syndrome as a cause of insulin resistance.

Serologic tests for antibody towards insulin yielded positive results for antibody specific for insulin (IgG and IgM) with antibody titre raised at 0.20nmol/L (reference range <0.02nmol/L) while IgE antibody to insulin was absent (analysed at Mayo Medical Laboratories, USA). Measurement of insulin levels after polyethylene glycol (PEG) testing showed reduced free insulin levels; 47% recovery while on glargine (Lantus) alone (pre-PEG insulin levels: 10.44mIU/L), and 36% recovery on aspart (Novorapid) insulin infusion (pre-PEG insulin levels:4.25mIU/L), suggesting impaired free-insulin level likely due to presence of antibodies binding to insulin.

When her rashes recurred after one year on insulin pump, skin biopsy was performed at the site of cutaneous reactions to insulin. Histology showed dermal oedema and a moderate amount of lymphohistiocytic infiltrate admixed with some eosinophils, acute inflammatory yield and fibrinous exudates around the superficial dermal vessels and hair follicles (**Figure 2E**), in keeping with our clinical diagnosis of insulin allergy. The onset of few hours after injection of insulin aspart pointed towards type III hypersensitivity. As the localised insulin reactions recurred also on insulin pump treatment, patch testing to the additive and consumables of insulin were performed. The patch tests were unremarkable to cresol, adhesive, plastic needle, and plastic cannula. The patient was also patch tested (Chemotechnique Diagnostics, Vellinge, Sweden) to the international standard series (including allergens such as nickel and cobalt), cosmetic series (including allergens such as 2-tert-butyl-4-methoxyphenol, 2,6-ditert-butyl-4-cresol and p-chloro-m-cresol) and methacrylate series with negative results for all.

## Therapeutic Intervention, Outcomes and Follow-up

Management of glycaemic control was challenging and required constant insulin titration upwards; at 1 year later her insulin carbohydrate ratio (ICR) was 1:2 and basal insulin glargine was 0.6-0.7 units/kg/day with split dosing. Her glycaemic control remained suboptimal with hyperglycaemia (especially post-prandial), high glycemic variability, and raised HbA1c ≈10% despite full adherence to MDI (**Figure 1**). Her TDD reached a peak of 2 units/kg/day when she consumed a moderate carbohydrate intake (100-200g/day). To improve post-prandial hyperglycaemia despite high ICR, she self-restricted her carbohydrate intake to 30g carbohydrate per meal (ICR 1:2) at 3 meals a day with similar diet plans daily. Despite efforts to adjust ICR, her glucose readings fluctuated widely even with the same carbohydrate portion, activity level and insulin dose.

Continuous subcutaneous insulin infusion (CSII) with aspart was started in view of persistent suboptimal glycemic control. This improved her glycaemic control in terms of glycaemic variability, postprandial hyperglycaemia, and improved HbA1c to ≈7% (**Figure 1**), reduction of ICR to 1:3 and reduction of total basal insulin dose to 0.4-0.5 units/kg/day. The low glucose suspend safety feature of Minimed Medtronic pump 640G^®^ was helpful to minimise hypoglycaemia however, the patient did not use the feature frequently because she did not like the additional attachment and cost of continuous glucose monitoring. At 1 year on insulin pump therapy, erythematous pruritic insulin injection reactions recurred and progressively worsened (**Figure 2C**). She was switched from insulin aspart to lispro or regular insulin (actrapid) on different occasions with similar hypersensitivity reactions and no improvement of clinical outcome. At 2-3 years after insulin pump initiation, she continued to experience tolerable cutaneous reactions at the sites of insulin administration resulting in an increasingly altered cosmesis of the abdominal skin (**Figure 2D**) and basal insulin requirement increased to 0.5-0.6 units/kg/day. When the patient switched back from insulin pump to MDI therapy with insulin glargine and aspart, there were still erythematous localised reactions. Her rashes remained tolerable with infrequent antihistamine use. Blood tests for full blood count and eosinophil levels were consistently normal throughout the years. Topical and oral steroids were considered but not used in this case due to lack of strong evidence for benefits of steroids while weighing against its side effects (7, 8).

## Discussion

This case is unique because the timeline of events suggests that insulin detemir triggered the event of insulin allergy. This was followed by rapid onset of complete insulin deficiency, and worsening insulin resistance. Exogenous insulin causing allergy is uncommon (1, 5) and immunological insulin resistance is rare (2). The very rapid-onset of DKA within few days of symptomatic hyperglycaemia with undetectable C-peptide levels and mostly negative for pancreatic autoantibodies, presents much similarity to a fulminant case of type 1 diabetes (9, 10). However, her pre-existing diagnosis of T2DM was not in line with the current definition of “fulminant type 1 diabetes” (10). To our knowledge, this is the first case report of insulin-dependent DM presenting with DKA which occurred after exposure to an exogenous insulin (detemir), and another rare report of a case of exogenous insulin allergy with concomitant immunological form of insulin resistance (11).

Detemir is an insulin analogue which differ from native insulin by the deletion of amino acid threonine B30 and addition of the saturated long-chain fatty acid myristic acid residue at B29 (12). Although all insulin analogues resemble human insulin, insulin analogues can cause immunogenicity leading to antibodies against insulin (2, 3) and insulin allergy (1, 5, 6, 11, 13). Among the long-acting insulin analogues, there are more reports of insulin detemir causing severe allergy (5, 6, 12), raising the question whether detemir is particularly immunogenic. Her clinical presentation did not fit into the usual DM classifications including T1DM, Latent Autoimmune Diabetes in Adult (LADA) and fulminant type 1 diabetes. She did not have ketosis-prone diabetes as her beta-cell failure was permanent. Whilst underlying aetiology of these conditions remain unclear, pathological immune reactions leading to pancreatic destruction have been postulated in T1DM and fulminant type 1 diabetes (9, 14), as well as checkpoint inhibitor-induced IDDM (15). Antibody testing is less diagnostic in Asian populations due to the lower prevalence of antibody-positivity in T1DM (14), while a minority of fulminant type 1 diabetes also have pancreatic autoantibodies (9). It is unclear what precipitated beta-cell failure in this patient.

Our patient possibly had immunological impaired insulin action and resistance leading to fluctuating hyperglycaemia and hypoglycaemia despite strict compliance to diet and insulin. Her insulin resistance could not be explained in total by her mild obesity. High-affinity antibodies towards insulin induce insulin resistance by causing low circulating free insulin levels whereas low-affinity antibodies cause hypoglycaemia due to delayed dissociation of the insulin from insulin-antibody complexes (2, 16). This phenomenon is referred as insulin antibody syndrome [IAS] when caused by non-insulin (e.g., methimazole) (17) and exogenous insulin antibody syndrome [EIAS]) when caused by exogenous insulin (2). The raised total antibody of IgG and IgM towards insulin and reduced free insulin level (post-PEG) (2, 16), supports our postulation. However, we are unable to prove the mechanisms because mechanistic and reaction kinetic studies to elucidate the affinity and capacity of the antibodies to insulin (16) were not available. Thus, it is debatable whether the insulin-antibody complex-induced resistance was at the subcutaneous level (i.e. subcutaneous insulin resistance syndrome [SIR]) (18) or systemic circulation level (EIAS) (2) or both, none of which is easily differentiable. The pathophysiology of SIR is also poorly understood; possible mechanisms include immune-mediated insulin resistance, enzymatic activity at subcutaneous sites, and insulin sequestration in adipose tissues (16, 18).

Insulin allergy is caused by type I (IgE-mediated), type III (antigen-antibody complexes) or type IV (delayed-type) hypersensitivity, of which type I is most commonly reported (1). The onset of symptoms for type I, III and IV occur within minutes, 2-6 hours, and 1-3 days later respectively. Detemir has been reported to cause type I, III and IV hypersensitivity reactions (1, 5, 12, 19). The raised antibodies towards insulin (IgG and IgM) are not specific for EIAS and could indicate type III hypersensitivity. Although skin prick testing to insulin is helpful, it is not always necessary for type III hypersensitivity, and there is concern of inducing serum sickness on re-introducing the allergenic insulin (1, 19). Hypersensitivity to insulin preparations including additives (e.g. zinc) and solvents (e.g. cresol) may mimic insulin allergy (1). In this case, the patient also had localised skin reactions to multiple various insulin formulations i.e. detemir, glulisine[apidra] (does not have zinc), aspart[novorapid], lispro[humalog] and regular insulin [actrapid]. Although these insulin formulations had cresol, she was patch tested negative for cresol. Other insulin-allergy mimics reported are due to allergens in insulin infusion devices (20), allergy to natural latex rubber antigens in insulin injection material (21) and faulty injection technique (22).

Avoiding insulin in the management of C-peptide negative subjects is not possible. Our literature review showed that insulin allergy in patients with T1DM have been reported in 16 cases (**Table 1**). Management of patients with T1DM with insulin allergy varies, with 7 cases switching the insulin type used (7, 8, 19, 25, 30–32), and 8 cases adopting the use of anti-histamines (7, 26–30, 32, 33). Where anti-histamines did not work or insulin therapy could not be switched, other measures are explored, with 5 cases adopting the use of insulin desensitisation with continuous subcutaneous insulin infusion (7, 11, 24, 28, 29), and 6 cases adopting the use of immunosuppression *via* steroids (7, 8, 23, 26, 29, 33), tacrolimus (30), rituximab (anti-CD20 monoclonal antibody) and IV immunoglobulin (11). Matheu et al. (29) in particular adopted both insulin desensitisation as well as immunosuppression strategies. The initial approach to management of insulin allergy was similar to approaches recommended by Heinzerling et al. (34) and Jacquier et al. (35), with switch to different insulin preparation and symptomatic therapy with anti-histamines being the first-line treatment before exploring other options such as inducing tolerance *via* continuous subcutaneous insulin infusion or specific immunotherapy with glucocorticoids. Other options that were successful after initial immunotherapy did not work or had complications include omalizumab, rituximab, mycophenolate mofetil, or colchicine and mercaptopurine (7, 8). However, use of immunomodulators is lacking strong clinical evidence and is associated with side effects e.g., mild hypersensitivity infusion reactions such as fever and chills, infections, and rituximab-associated progressive multifocal leukoencephalopathy (36). Leonet et al. attempted a vascularised whole pancreas transplant as last resort (30).

**Table 1:** Literature review of presentation and management of patients with type 1 diabetes mellitus with insulin allergy.

	Year of Publication, Place	Age (yr), gender	Duration from diagnosis of T1DM to presentation	Insulin therapy at allergy presentation	Type of hypersensitivity and Presenting features	Intervention	Outcome and follow up
1	Grant et. al., 1986 (23) United States of America	16 Female	6 years	1 month of Continuous Subcutaneous Insulin Infusion: Lilly Humulin regular insulin	Biphasic-type insulin reaction (wheal and flare followed by late reaction at 6 – 12 hr) Persistent induration, wheal, and flares reactions at site of continuous subcutaneous insulin infusion therapy	Incorporation of methylprednisolone to human insulin (0.04mg of methylprednisolone/1U of insulin). Lower doses at 0.02mg of methylprednisolone/1U of insulin also found to be successful.	After several months, methylprednisolone gradually discontinued, and reactions did not recur.
2	Chng et. al., 1995 (24) Singapore	22 Male	11 months	3 weeks of Humulin R and N	Type I hypersensitivity Immediate local reaction which settles within 20 mins. Months after onset of local reaction, started to develop generalised urticaria which resolved spontaneously in 1hr	Slow desensitisation protocol with 3 doses of Humulin R followed by modified rapid desensitisation at hourly intervals on day 5. He continued to have small local reactions (<10mm wheal size) and was discharged 13 days after desensitisation. Two weeks after discharge, he relapsed and was advised to reduce Humulin R and Humulin N dose.	Repeated small local reactions of less than 10mm diameter at week 6
3	Blanco et. al., 1996 (25) Spain	20 Male	1 year	1 year of Neutral Protamine Hagedorn insulin (Insulatard Novolet)	Type I hypersensitivity Pruritus, wheal, flare over injection site, followed by generalised flushing, pruritus, dyspnoea, wheezing. On the third episode, progressed to hypotension	Test dosed to regular human insulin and lente human insulin where he showed perfect tolerance. Discharged with one dose of lente human (rDNA) insulin.	No new reactions at 1 year of follow-up
4	Silva et. al., 1997 (26) Brazil	33 Female	25 years	3 years of human insulin	Type III hypersensitivity Small, localised, subdermal, tender and painful non-erythematous nodules with central hematoma at injection sites, occurs 6 – 8 h after insulin injection and lasts for 48h Also had mild, generalised urticaria for 8 years, partially controlled with oral antihistamines whilst on mixed pork-beef insulin	Cetirizine was started but reaction persisted after a month. Prednisone 40mg/day was associated to the regimen of short acting human insulin and oral antihistamine.	After 4 months of treatment, urticaria and nodules disappeared
5	Gonzalo et. al., 1998 (27) Portugal	32 Female	3 months	45 days of Actrapid and Mixtard insulin	Type I hypersensitivity Local reactions at injection sites with pruritus, wheal and flare 5 mins after every injection. Reached a diameter 8- 10cm after 1hr, resolved within 1–2hr, followed by an indurated lesion that lasted for 48hr. Skin biopsy showed a perivascular infiltrate consisting of lymphocytes, monocytes, eosinophils, mild oedema affecting the superficial dermis, slight oedema of endothelial cells	Cetirizine 10mg/24H	Persistent local reactions, unable to stop cetirizine
6	Sola-Gazagnes et. al., 2003 (28) France	21 Female	4 years	4 months of Semisynthetic human insulin	Type I IgE-mediated hypersensitivity Nettle rash involving injection sites beginning < 5 mins after each injection, subsided after 3 -4H	Reaction persisted despite H1 antihistamine treatment. Desensitisation with low dose insulin not appropriate due to patient’s strict insulin requirements. Switched to continuous low-dose subcutaneous insulin lispro infusion with external insulin pump for desensitisation and treatment of diabetes. Boluses were replaced with temporarily increased basal rates to avoid potential allergy reactivation. Usual oral cetirizine was maintained	No local reaction at insertion site of catheter or elsewhere. Cutaneous allergy resolved, premeal boluses introduced and antihistamine treatment stopped at 1 year.
7	Darmon et. al., 2005 (19) France	31 Male	20 years	6 hours after starting insulin detemir + aspart Previously was on regime of glargine (Lantus) + aspart (Novorapid)	Type III hypersensitivity Subcutaneous small, subdermal, non-pruriginous, slightly painful non-erythematous nodule with central hematoma 6H after first injection of detemir	Switched back to previous regime glargine plus aspart	The nodules spontaneously disappeared in 48H after switching back to glargine and aspart
8	Matheu et. al., 2005 (29) Spain	25 Male	2 years	Neutral Protamine Hagedorn insulin of unspecified duration 3 months of Human regular insulin	Type I hypersensitivity Wheals and flare local reactions to Neutral protamine Hagedorn Immediate wheals and flare reactions far from insertion site of catheter, intensive pruritus on palms and soles, dyspnoea, subcutaneous nodules to Human regular insulin	Methyl-prednisolone (15mg/day) and hydroxyzine incorporated and daily insulin requirement increased up to 2.4U/kg/day. Controlled re-exposure with bolus of regular insulin caused non-tender swelling with flares (5–6cm diameter) far from insertion of catheter. Skin biopsy revealed subcutaneous oedema with infiltrated cells including eosinophils. Desensitisation performed with insulin Aspart via subcutaneous insulin pump with methyl-prednisolone at 30mg/day. Hydroxyzine was stopped.	Six months after end of desensitisation, daily insulin requirement decreased to 0.8U/kg/day and methyl-prednisolone dose decreased to 2mg/48H
9	Léonet et. al., 2006 (30) Belgium	29 Male	14 years	Actrapid insulin	Type I hypersensitivity Malaise, tremor, tachycardia, burning sensation, nervosism, visual troubles, dizziness few minutes after each administration, after 3 years, starting becoming breathless with short episodes of unconsciousness	Given anti-histamine therapy, switched from Actrapid to Insulin Paraben NovoNordisk, received a HLA-DR semi-identical compatible blood transfusion along with tacrolimus which all did not work. Received a vascularised whole pancreas transplant resulting in complete resolution	Well at 24 months after transplantation
10	Sola-Gazagnes et. al., 2007 (12) France	25 Male	7 years	Detemir insulin	Type I hypersensitivity Erythematous papulae	Either continuing insulin detemir and was able to stop antihistamines a few weeks later or returned to previous treatment with rapid-acting insulin infusion	Not reported
11	Yong et. al., 2009 (7) United Kingdom	50 Female	Not specified	Various insulin preparations	Type I hypersensitivity Generalised urticarial rash resulting in excoriation, bleeding, disrupted sleep	Various insulin preparations, anti-histamines, insulin desensitisation were not successful. Prednisolone alone provided symptomatic relief but brought about complications and required higher doses. Rituximab administered to reduce IgE levels so omalizumab can be given, mycophenolate mofetil after 4 weeks	Remained asymptomatic at 9 months while receiving 2mg of prednisolone/day with falling HbA1c levels. Serum IgE levels increased which was expected after initiation of omalizumab which binds IgE to form drug-IgE complexes which diminish level of bioavailable IgE
12	Watanabe et. al., 2016 (31) Japan	28 Female	4 years	Continuous Subcutaneous Insulin Infusion of Lispro insulin	Type I hypersensitivity Redness, swelling, itchiness, induration over cannula insertion site	Switch to Continuous Subcutaneous Insulin Infusion with insulin glulisine	Milder allergic reaction, no redness and swelling at cannula insertion site. IgE antibodies specific to human insulin also decreased at week 8
13	Murray et. al., 2017 (8) United States of America	23 Female	Several years	18 months of lispro and glargine insulin	Type III hypersensitivity Pruritic rash 30 mins after each injection, becoming progressively swollen and painful over 2 – 3H which resolved on their own after several days. Large, tender, indurated, purpuric plaques over sites of prior injection (histo showed leukocytoclastic vasculitis)	Methylprednisolone initially lessened the reaction but failed with higher insulin doses. Slow infusion glulisine (Apidra) then selected which was initially well tolerated, but skin reaction developed on day 7. Subsequently tolerated low-dose glargine with colchicine and mercaptopurine	Persistent mild reactions that were dose dependent at 6 months after admission
14	Mastrorilli et. al., 2017 (32) Italy	9 Female	1 week	Glargine and lispro insulin	Type I hypersensitivity Generalised immediate urticaria after insulin glargine injections	Oral antihistamine and switch insulin glargine to insulin degludec which caused generalised urticaria within minutes which recovered after treatment with oral anti-histamine. After skin prick and intradermal test of insulin detemir showed negative results, insulin detemir was administered which did not trigger any allergic reaction.	No adverse reactions to insulin detemir at 6 months follow-up
15	Harvey et. al., 2020 (11) United Kingdom	12 Female	2 years	4 months of Insulin glargine and aspart	Type III hypersensitivity Raised, red, painful, pruritic subcutaneous nodules hours after insulin administration which lasted 4 days before resolving.	Antihistamines produced minimal improvement with flares at distant injection sites. Subcutaneous insulin injections of different insulin products (Actrapid, Humulin S, Porcine neutral, Bovine neutral, Hypurin bovine lente, Aspart, Detemir, Glargine, Glulisine and Lispro insulin) were tried, but she reacted with troublesome local reactions with tender red subcutaneous nodules with widespread lipoatrophy at older injection sites. Hyposensitisation using continuous SC insulin infusion which was initially well tolerated but developed cutaneous reactions on day 2. After rituximab was given, there was redness at injection sites but no discomfort or nodule formation. IV immunoglobulin was given to boost the immune depletion	After IV immunoglobulin was given, patient well with no inflammatory reactions on injection. However, subsequently, her glycemic control worsened and admission for DKA. 6 years later, she did not have skin reactions to insulin and had reduction of insulin requirement.
16	Aujero et. al., 2011 (33) Korea	39 Female	Not specified	2 weeks of Insulin detemir	Serum-sickness type III reaction Urticarial rash 2 weeks after commencing insulin detemir, which abated after 5-day prednisolone taper. 3 days after completing the taper, facial edema, fever, arthralgia and urticaria developed. Prednisolone taper allowed for temporary respite, but rash recurred after completion. Pruritic rash, joint pain, fatigue, multiple urticarial plaque involving trunk and arms with blanching erythema at sites of prior lesions Skin biopsy showed superficial and deep perivascular dermatitis.	Treated with fexofenadine, cetirizine, 4-week prednisolone taper	Symptoms and examination findings resolved with normalisation of acute phase reactants

In conclusion, the workup and management of patients with insulin hypersensitivity is challenging. More studies are required to understand the pathophysiology of exogenous insulin-induced adverse reactions in order to develop safe and effective treatment.

## Data Availability Statement

The original contributions presented in the study are included in the article/supplementary material. Further inquiries can be directed to the corresponding author.

## Ethics Statement

Written informed consent was obtained from the individual for the publication of any potentially identifiable images or data included in this article.

## Author Contributions

WJL is the physician taking care of the patient, wrote the case report and was involved in the final approval of the paper. CBT and PYT drafted the case report and performed literature research. SXL was involved in care of and assessment of the patient’s dermatology condition. JK, SCL and BB were involved in the critical revision of the paper. JGT and SFA were involved in the laboratory analyses. SHT was involved in analyzing the histological slides. TLT and ET are the physicians involved in taking care of the patient. All authors contributed to the writing of the article and approved the submitted version.

## Conflict of Interest

The authors declare that the research was conducted in the absence of any commercial or financial relationships that could be construed as a potential conflict of interest.

## Publisher’s Note

All claims expressed in this article are solely those of the authors and do not necessarily represent those of their affiliated organizations, or those of the publisher, the editors and the reviewers. Any product that may be evaluated in this article, or claim that may be made by its manufacturer, is not guaranteed or endorsed by the publisher.
